# Electronic Properties of a New All-Inorganic Perovskite TlPbI_3_ Simulated by the First Principles

**DOI:** 10.1186/s11671-017-2015-y

**Published:** 2017-03-29

**Authors:** Zhao Liu, Ting Zhang, Yafei Wang, Chenyun Wang, Peng Zhang, Hojjatollah Sarvari, Zhi Chen, Shibin Li

**Affiliations:** 1 State Key Laboratory of Electronic Thin Films and Integrated Devices, School of Optoelectronic Information, University of Electronic Science and Technology of China (UESTC), Chengdu, Sichuan 610054 China; 2 Department of Electrical & Computer Engineering and Center for Nanoscale Science and Engineering, University of Kentucky, Lexington, KY 40506 USA

**Keywords:** All-inorganic perovskite, TlPbI_3_, CsPbI_3_, First principles

## Abstract

All-inorganic perovskites have been recognized as promising photovoltaic materials. We simulated the perovskite material of TlPbI_3_ using ab initio electronic structure calculations. The band gap of 1.33 eV is extremely close to the theoretical optimum value. Compared TlPbI_3_ with CsPbI_3_, the total energy (−3980 eV) of the former is much lower than the latter. The partial density of states (PDOS) of TlPbI_3_ shows that a strong bond exists between Tl and I, resulting in the lower total energy and more stable existence than CsPbI_3_.

## Background

Hybrid organic–inorganic halide perovskites ABX_3_ (A is an organic cation, B is Pb or Sn, and X is a halide) have been widely used as solar cells and attracted enormous interest due to the low-cost and simple solution process for extensive production in the field of photovoltaic (PV) applications. The rapid rise of hybrid organic–inorganic perovskite solar cells has seen photoelectric conversion efficiencies rise from 3.8% [1] to 21.1% [2] in less than 6 years, although the fact that the perovskite absorber layers are subject to degradation because of heat and humidity. To overcome these issues, numerous investigations on enhancing the efficiency [3, 4] and long-term stability [5, 6] have been performed for years [7–12], and now, the perovskite with all-inorganic structure is a primary focus [13]. For solar cells, an appropriate band gap will give a satisfactory efficiency. And the band gap should be narrow enough to absorb a broad solar spectrum from near infrared to visible light. The open-circuit voltage Voc is always lower than the band gap energy because thermodynamic detailed balance requires the cell to be in equilibrium with its environment, which indicates that there is spontaneous light emission from the cell. Considering the two factors, the cubic cesium lead iodide (CsPbI_3_) is a promising candidate for PV devices. Reference [14] reported the maximum efficiency occurs for a semiconductor with a band gap of 1.34 eV and is 33.7%.

**Fig. 1:**
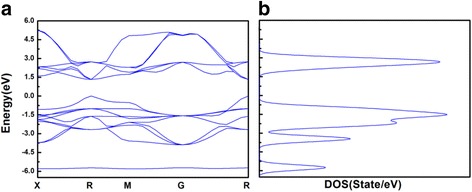
**a**–**b** The band structure and density of states of TlPbI_3_

**Fig. 2:**
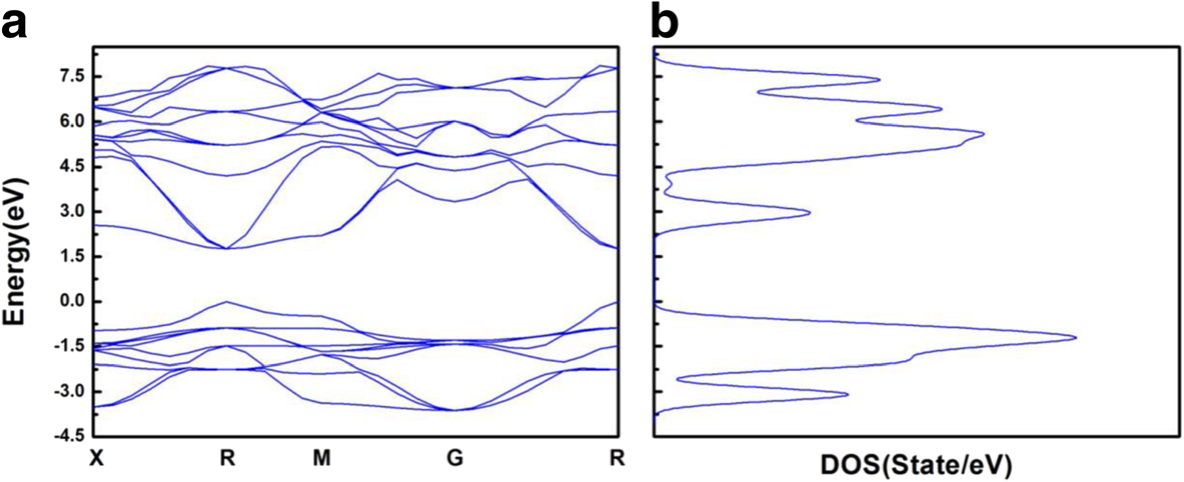
**a**–**b**The band structure and density of states of CsPbI_3_

The outer electron configuration of the thallium atom is [Xe]4f^14^5d^10^6s^2^6p^1^, which has two valence states of +1 and +3, +1 valence compounds are more stable than +3 [15]. In this paper, we simulated the perovskite material of TlPbI_3_ with a band gap of 1.33 eV using ab initio electronic structure calculations based on the Density Functional Theory (DFT), and the band gap is extremely closer to the theoretical optimum value than CsPbI_3﻿_ (Figs. 1 and 2). Compared TlPbI_3_ with CsPbI_3_, the total energy (−3980 eV) of the former is much lower than the latter. The partial density of states (PDOS) of TlPbI_3_ shows that a strong bond exists between Tl and I, resulting in the lower total energy and more stable existence than CsPbI_3_. Besides, we calculated the carrier concentration and found both the two materials indicate similar carrier concentration ranged from −20 to 50 °C.

### Methods

We employed ab initio electronic structure calculations with DFT and the generalized gradient approximation (GGA) [16] put forward by Perdew–Burke–Ernzerhof (PBE) [17]. We used plane-wave basis sets and pseudopotentials. Kohn and Hohenberg [18] suggested that the real density of electrons would lead to a quite tiny functional value. Thus, Shan and Kohn optimized and put forward the density functional theory again, namely Kohn–Sham equations (KS equation) [19]:

1 $$ \left[-\frac{1}{2}{\nabla}^2+{\nu}_{\mathrm{ext}}\left(\overrightarrow{r}\right)+{\nu}_{\mathrm{H}}\left(\overrightarrow{r}\right)+{\nu}_{\mathrm{xc}}\left(\overrightarrow{r}\right)\right]{\varphi}_i={\varepsilon}_i{\varphi}_i $$

Formula (1) represents the motion of the electrons in the molecular system. Where, ${\nu}_{\mathrm{ext}}\left(\overrightarrow{r}\right)=-{\displaystyle \sum_{\alpha =1}^{\mathrm{N}}\frac{Z_{\alpha}}{\left|\overrightarrow{r}-\overrightarrow{r_{\alpha}}\right|}}$ is the interaction between electrons and atoms, namely external potential. ${\nu}_{\mathrm{H}}\left(\overrightarrow{r}\right)={\displaystyle \int \frac{\rho \left({\overrightarrow{r}}^l\right)}{\left|\overrightarrow{r}-{\overrightarrow{r}}^l\right|} d{\overrightarrow{r}}^l}$ stands for the interaction potential between electrons. ${\nu}_{\mathrm{xc}}\left(\overrightarrow{r}\right)$ is the functional differential of exchange-correlation energy. *E*_xc_. ${\nu}_{\mathrm{xc}}\left(\overrightarrow{r}\right)=\frac{\delta {E}_{\mathrm{xc}}\left[\rho \left(\overrightarrow{r}\right)\right]}{\delta \rho \left(\overrightarrow{r}\right)}$ represents the exchange-correlation potential. The effective potential ${v}_{\mathsf{eff}}={v}_{\mathrm{xtf}}+{v}_H+{v}_{\mathrm{xc}}$ is mainly determined by electron density, which can be obtained by KS equation. Obviously, the equation can be solved by self-consistent field equations (SCF) if we know the exchange-correlation energy *E*_xc_. After obtaining the self-consistent convergence charge density *ρ*_0_, the ground-state energy of the system can be expressed as [20]:

2 $$ {E}_0={\displaystyle \sum_{i=1}^N{\varepsilon}_i}-\frac{1}{2}{\displaystyle \int {\displaystyle \int \frac{\rho_0\left(\overrightarrow{r}\right){\rho}_0\left({\overrightarrow{r}}^l\right)}{\left|\overrightarrow{r}-{\overrightarrow{r}}^l\right|} d\overrightarrow{r} d{\overrightarrow{r}}^l}}-{\displaystyle \int {v}_{\mathrm{xc}}\left(\overrightarrow{r}\right){\rho}_0\left(\overrightarrow{r}\right) d\overrightarrow{r}+{E}_{\mathrm{xc}}\left[{\rho}_0\left(\overrightarrow{r}\right)\right]} $$

*ε*_*i*_ is the eigenvalue of Eq (1):

3 $$ {\varepsilon}_i=\left\langle {\varphi}_i\left|-\frac{1}{2}{\nabla}^2+{v}_{\mathsf{eff}}\right|{\varphi}_i\right\rangle $$

In theory, the KS equation derived from DFT should be accurate [21]. But in the specific case, as *E*_xc_ is a function associated with the single electron density $\rho \left(\overrightarrow{r}\right)$, it is necessary to find a function that can replace the single electron density. We can solve a set of *φ*_*i*_ by taking *v*_xc_ into the KS equation. Then a new *v*_xc_ can be calculated with this *φ*_*i*_. Finally, we submit it into KS equation and solve. Repeat the iteration until a certain accuracy. The key problem is to find the appropriate exchange correlation energy *E*_xc_. In the case of different calculation methods of exchange correlation energy *E*_xc_, a series of DFT models have been reported [22]. The GGA method is more accurate because it has been combined with inhomogeneous electron gas to obtain ${E}_x^{B88}$, ${E}_x^{\mathrm{LYP}}$ and other parameters [23].

To calculate out carrier concentration, we must know the effective mass of electron, expressed by the following formula [18].

4 $$ {m}_e^{*}={\hslash}^2{\left[\frac{d^2 E}{d{ k}^2}\right]}^{-1} $$

As expressed in formula (4), effective mass of holes and electrons can be obtained by calculating the two derivatives of valence-band maximum and conduction-band bottom. Finally, the carrier concentration is obtained as follows:

5 $$ {n}_i=\left(2.510\times {10}^{19}\right){\left(\frac{m_n^{*}}{m_0}\cdot \frac{m_p^{*}}{m_0}\right)}^{\frac{3}{4}}{\left(\frac{T}{300}\right)}^{\frac{3}{2}}{e}^{\frac{-{E}_g}{2 T}} $$

$\frac{m_n^{*}}{m_0}$ is the effective mass of electron, obtained by:

6 $$ \frac{m_n^{*}}{m_0}=\frac{{\left(\frac{6.626\times {10}^{-34}}{a\times {10}^{-10}}\right)}^2}{X_0\times 1.6\times {10}^{-19}\times 9.109\times {10}^{-31}} $$

of which, *X*_0_ is the two derivatives of conduction-band bottom. *a* is the lattice constant. Instead of conduction-band bottom by valance-band maximum, the formula (6) is often applied to solve the effective mass of hole $\frac{m_p^{*}}{m_0}$ [24].

The Brillouin zone was sampled with a 2 × 2 × 2 k-point set and built by 2 × 2 × 2 supercell. The simulated models using 6s^2^4f^14^5d^10^ and 5s^2^4d^10^5p^6^ as valence electrons for Tl and Cs, respectively, are carried out. Firstly, we use the ultrasoft pseudopotentials to optimize the Pm3m structures of both TlPbI_3_ and CsPbI_3._ Then, we calculate the equilibrium volume and proper values of the lattice constants. After optimizing the crystalline structure, we calculate the total energy, band structure, density of states, and carrier concentration for two kinds of materials in the last.

## Results and Discussion

Optimizing the geometry of TlPbI_3_ and CsPbI_3_, we have simulated that the lattice constants are 6.2621 Å and 6.3225 Å, respectively. The bond distances and bond angels calculated are presented in Table 1.

**Table 1:** The bond distances and bond angels of TlPbI_3_ and CsPbI_3_

	*X* = Tl	*X* = Cs
X-Pb	5.423 Å	5.475 Å
X-I	4.428 Å	4.471 Å
Pb-I	3.131 Å	3.161 Å
X-Pb-I	54.736I	54.736I

The total energies of TlPbI_3_ and CsPbI_3_ are −3979.94 − 3154.36 eV independently. The lower total energy means the better stability. Thus, a conclusion that TlPbI_3_ has better stability than CsPbI_3_ is summarized theoretically.

**Fig. 3:**
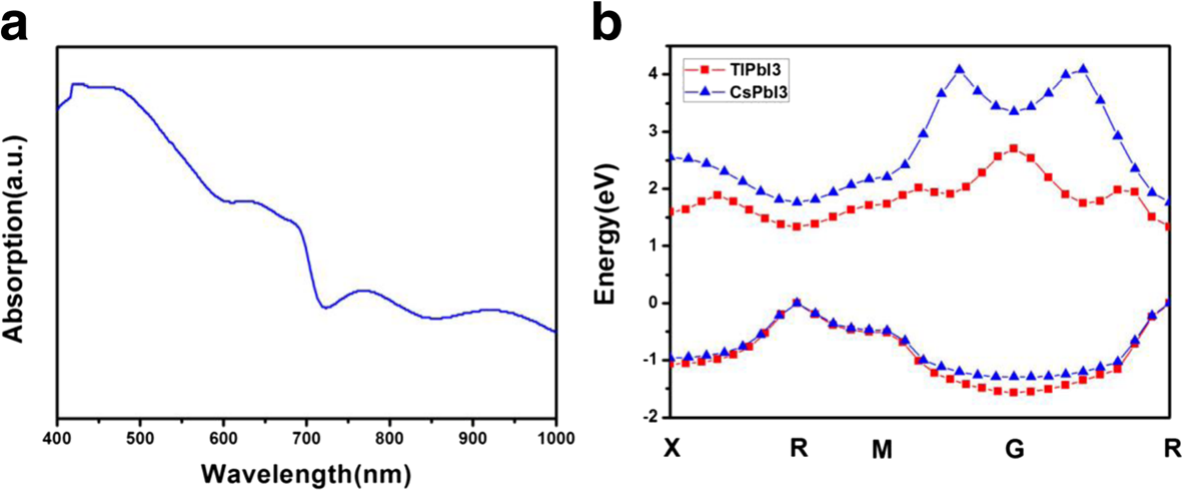
**a** is the absorption spectrum of CsPbI_3_ measured by experiment. **b** is the structures of valence-band maximum and conduction-band minimum of TlPbI_3_ and CsPbI_3_

The band gap of both semiconductors are calculated out 1.763 and 1.331 eV, respectively. The absorption spectrum of CsPbI_3_ measured by the experiment (Fig. 3a) shows the optical band gap is 1.73 eV, which is close to the simulated value. And the absorption decreases above the wavelength of ~716 nm. The outcomes further indicate that the model we simulated are correct. Both of their Fermi levels are extremely close to the valence-band maximum, meaning that they are p-type semiconductors. The band gap of TlPbI_3_ is quite close to the perfect semiconductor reported in reference [14]. If we can fabricate the solar cells with this material, a high efficiency will be obtained. However, the devices based on TlPbI_3_ are limited to simulate because of the toxicity.

Here, we select the valence-band maximum and conduction-band minimum for further analysis. As shown in Fig. 3b, the curvature of energy band in TlPbI_3_ is less than that in CsPbI_3_. The conduction band of TlPbI_3_ is relatively smooth and conducive to receive electron from valence band, enhancing the existence of carries.

**Fig. 4:**
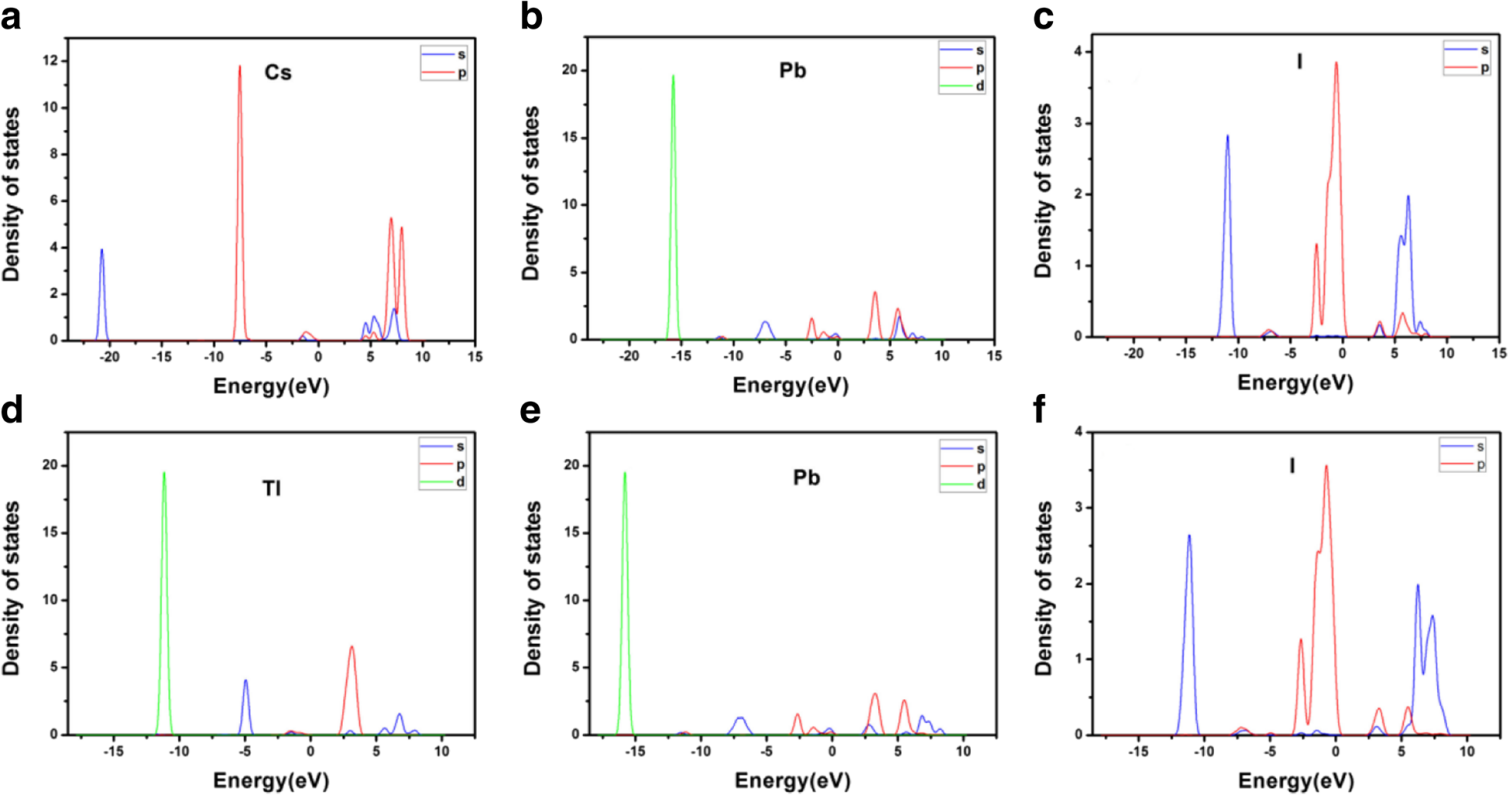
**a**–**c** are the partial density of states (PDOS) in CsPbI_3_. **d**–**f** are the partial density of states (PDOS) in TlPbI_3_

According to the molecular orbital theory [25], the corresponding to the bond or anti-bond orbitals are formed by the more gentle part of the band curve. As shown in Figs. 1 and 2, if the peak of DOS curve is quite sharp, the corresponding energy-band curve is smooth; if the peak of DOS curve is relatively flat, the energy-band curve is relatively curved. So it can be deduced that the molecular orbitals are consistent with the peaks of the DOS graph. The peak height in the PDOS diagram (shown in Fig. 4) reflects the number of electrons contributing to this peak. If the PDOS of two different atoms has the resonance peaks in the range of same energy, it means the two atoms have already bonded. However, it cannot be determined that whether the band or anti-band are formed by analyzing the PDOS without further experiments. When the existence of resonance peaks are caused by the interaction of several atoms, we cannot distinguish that the bonds are formed by the two specific atoms. The conclusion does not affect our analysis.

As illustrated in Fig. 4a–c, the conduction-band minimum of CsPbI_3_ is mainly composed by 6p state of Pb, and the valence-band maximum is contributed by 5p states of I. In Fig. 4d–f, the bottom of the conduction band of TlPbI_3_ is mainly composed by both 6p states of Tl and 6p states of Pb, and the top of the valence band is contributed by 5p states of I. As presented in Fig. 4d–f, Tl and I have a strong resonance peaks between −12 and −10 eV, resulting in a deep level state. It also explains why TlPbI_3_ is more stable than CsPbI_3_.

**Fig. 5:**
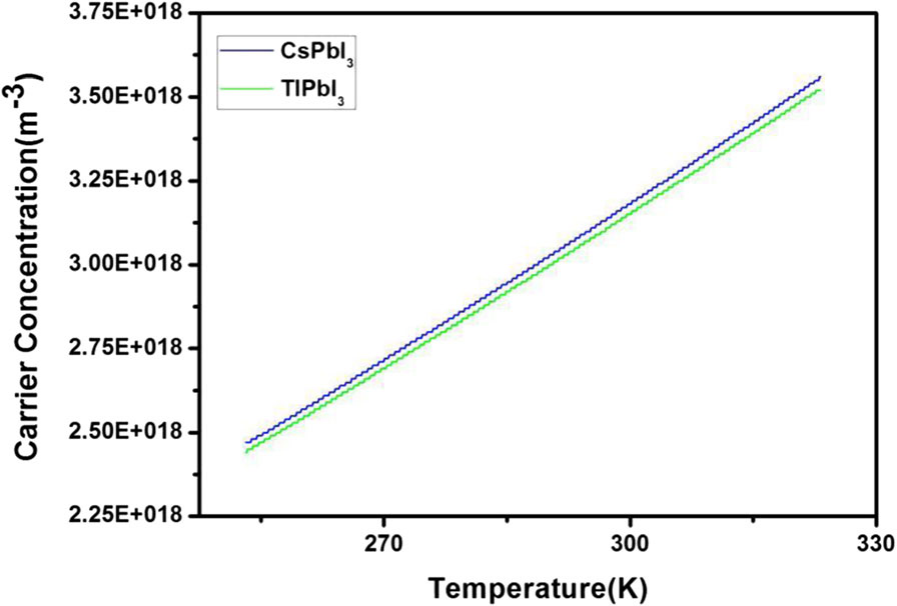
The relationship between temperature and carrier concentration

Finally, carrier concentration are calculated by formulas (5) and (6). The relationship between temperature and carrier concentrations of both TlPbI_3_ and CsPbI_3_ is shown in Fig. 5. The carrier concentration of TlPbI_3_ is slightly less than that of CsPbI_3_ because the electronegativity of Tl (2.04) atom is larger than that of Cs (0.79) atom [26]. A larger electronegativity leads to a larger ionic bond component and stronger polarity, enhancing the attraction between electrons.

## Conclusions

We simulated the perovskite material of TlPbI_3_ with a band gap of 1.33 eV using ab initio electronic structure calculations and the band gap is extremely close to the theoretical optimum value. Compared TlPbI_3_ with CsPbI_3_, the total energy (−3980 eV) of the former is much lower than the latter. The partial density of states (PDOS) of TlPbI_3_ shows that a strong bond exists between Tl and I, resulting in the lower total energy and more stable than CsPbI_3_. Besides, we calculated the carrier concentration and found both the two materials have similar carrier concentration ranged from −20 to 50 °C.
